# Dissecting Structural and Functional Diversity of the Lantibiotic Mersacidin

**DOI:** 10.1016/j.chembiol.2009.03.011

**Published:** 2009-05-29

**Authors:** Antony N. Appleyard, Shaila Choi, Daniel M. Read, Ann Lightfoot, Steven Boakes, Anja Hoffmann, Ian Chopra, Gabriele Bierbaum, Brian A.M. Rudd, Michael J. Dawson, Jesus Cortes

**Affiliations:** 1 Novacta Biosystems Ltd., BioPark Hertfordshire, Welwyn Garden City, Hertfordshire AL7 3AX, UK; 2 Antimicrobial Research Centre and Institute of Molecular and Cellular Biology, University of Leeds, Leeds LS2 9JT, UK; 3 Institut für Medizinische Mikrobiologie und Immunologie, Universität Bonn, 53105 Bonn, Germany

**Keywords:** CHEMBIO, MICROBIO

## Abstract

Mersacidin is a tetracyclic lantibiotic with antibacterial activity against Gram-positive pathogens. To probe the specificity of the biosynthetic pathway of mersacidin and obtain analogs with improved antibacterial activity, an efficient system for generating variants of this lantibiotic was developed. A saturation mutagenesis library of the residues of mersacidin not involved in cycle formation was constructed and used to validate this system. Mersacidin analogs were obtained in good yield in approximately 35% of the cases, producing a collection of 82 new compounds. This system was also used for the production of deletion and insertion mutants of mersacidin. The outcome of these studies suggests that this system can be extended to produce mersacidin variants with multiple changes that will allow a full investigation of the potential use of modified mersacidins as therapeutic agents.

## Introduction

The lantibiotics are a diverse group of highly modified peptides produced by Gram-positive bacteria sharing the common feature of (methyl)lanthionine ring structures (Sahl and Bierbaum, 1998). Lantibiotics are derived from ribosomally synthesized peptides that are subject to posttranslational modifications. Serine or threonine residues are dehydrated to dehydroalanine (Da) or dehydrobutyrine (Db), respectively. These residues undergo Michael addition with the thiol group of cysteine residues to form the characteristic (methyl)lanthionine rings. Apart from these distinctive rings, some lantibiotics also contain other modified residues or ring structures, such as the 2-thioethenamine moiety in mersacidin (Chatterjee et al., 1992b) or epidermin (Allgaier et al., 1986), the lysinoalanine ring and hydroxyaspartate in cinnamycin (Kessler et al., 1987), d-alanine in lactocin S (Skaugen et al., 1994) and lacticin 3147 (Ryan et al., 1999), an N-terminal oxobutyryl group in Pep5 (Kellner et al., 1989) and lacticin 3147-A2 (Ryan et al., 1999), and dihydroxyproline and chloro-tryptophan in microbisporicin (Castiglione et al., 2008).

The biosynthetic pathways for a number of lantibiotics have been cloned and sequenced, and functions have been inferred for the genes on the basis of homology with genes of known function, gene disruptions, and enzymatic studies. As with other antibiotics or secondary metabolites, the biosynthetic genes are clustered together. The lantibiotic biosynthetic genes have been given the generic locus name *lan.* They are specifically assigned *lanA* for the gene encoding the lantibiotic prepeptide, *lanB* for the gene encoding the enzyme responsible for serine or threonine dehydration and *lanC* for the gene responsible for cycle formation. In some cases, the activities of LanB and LanC are housed in a bifunctional enzyme encoded by *lanM* (Sahl and Bierbaum, 1998). The products of the *lanP*, *lanT*, and *lanH* genes are involved in processing and/or transport of the prepeptide, and *lanI*, *lanE*, *lanF*, and *lanG* encode for immunity or resistance. Additional genes specific for particular features of some lantibiotics have been identified as well, such as *lanD*, which is responsible for the oxidative decarboxylation of the C-terminal cysteine of epidermin (*epiD*) (Kupke et al., 1992) or mersacidin (*mrsD*) (Majer et al., 2002).

**Figure 1:**
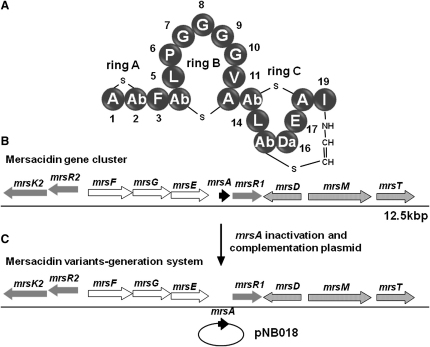
The Structure of Mersacidin and Its Gene Cluster (A) Primary structure of mersacidin indicating the sequence and thioether bridge pattern. (B) Organization of the mersacidin biosynthetic gene cluster. (C) Engineered system for generating mersacidin mutants. Da, dehydroalanine; Ab, 2-aminobutyrate.

Mersacidin is a lantibiotic produced by *Bacillus* sp. HIL Y-84,54728 at the late stages of growth and stationary phase (Chatterjee et al., 1992a). The biosynthetic gene cluster contains 10 genes spanning 12.3 kbp. Mersacidin is a tetracyclic peptide (Figure 1) containing 20 amino acid residues and is encoded by *mrsA* as a prepeptide of 68 residues (Bierbaum et al., 1995). All the genes in the cluster have been sequenced and the functions have been identified (Altena et al., 2000). The mersacidin prepeptide MrsA is decarboxylated and oxidized by MrsD, producing a 2-thioethenamine functionality at the C-terminal cysteine (Majer et al., 2002). This modified MrsA is proposed to be the substrate for MrsM that produces the tetracyclic structure, which is processed and secreted by MrsT as the final bioactive compound. There is a regulatory gene, *mrsR1*, which is responsible for controlling the synthesis of MrsA (Guder et al., 2002), and a two-component system, *mrsR2/mrsK2*, which encodes for the sensor and kinase involved in the induction of the immunity genes *mrsE, mrsF*, and *mrsG* (Guder et al., 2002).

Mersacidin has antibacterial activity against Gram-positive pathogens; like vancomycin, it binds to the peptidoglycan precursor lipid II (Brotz et al., 1995; Brotz et al., 1997). The results of lipid II binding experiments with mersacidin in the presence of vancomycin suggest that mersacidin binds to a different part of the molecule (Brotz et al., 1998); hence, no cross-resistance has been observed. Significantly, in vivo studies have shown that mersacidin administered subcutaneously cures systemic methicillin-resistant *Staphylococcus aureus* (MRSA) infections in mice and abscesses in rats at concentrations comparable to vancomycin (Chatterjee et al., 1992b). Further studies have shown that mersacidin also eradicates MRSA in a mouse rhinitis model (Kruszewska et al., 2004). The activity of mersacidin against MRSA makes it a potential candidate for development as a therapeutic agent; therefore, information on the key residues that contribute to the bioactivity and information on the flexibility of the biosynthetic pathway would be very valuable for the design of new molecules with improved properties. To probe the flexibility of the mersacidin biosynthetic pathway, a systematic mutagenesis study of the gene encoding for mersacidin and analysis of the fermentation products of the mutants was performed, and the results are presented below.

## Results

### Construction of an Efficient System for Production of Mersacidin Variants

A method for the construction of mersacidin variants has been reported by Szekat et al. (2003). Using this method, it was demonstrated that it is possible to generate variant mersacidin molecules; however, this approach is laborious and involves steps that are not suitable for obtaining large numbers of mutants. To simplify the genetic manipulations required for the construction of libraries of mersacidins, a *trans* complementation system was developed. This system consists of an inactive *mrsA* mutant of the mersacidin producer organism and a complementing shuttle plasmid capable of efficiently expressing *mrsA* or its mutants. A simplified transformation procedure to deliver this plasmid into the host by electroporation of demethylated DNA was developed, facilitating the overall procedure and making possible the generation of a large number of mutant strains.

### Host Strain for Mersacidin Libraries

A mutant of the mersacidin producer strain called *Bacillus* sp. TTEX, wherein the GAA codon of the fourth amino acid of MrsA was substituted for the stop codon TAA, was used as a host (Schmitz et al., 2006). This mutant does not produce MrsA and, consequently, does not produce mersacidin. All the other genes in the mersacidin pathway are functional.

### Construction of a Complementation and Expression Plasmid for *mrsA*

A PCR product from base 4836 to base 5249 of the mersacidin gene cluster (accession number AJ250862) containing the promoter and leader peptide encoding sequence of *mrsA* was obtained using the oligonucleotides jc7 and jc8 and the plasmid pMER1 (Altena et al., 2000) as a template. The PCR product was ligated to SmaI-digested pUC18. A plasmid with the expected sequence and ligated in the orientation such that the insert can be excised by digesting with EcoRI was selected and designated pNB013. Plasmid pNB013 was digested with EcoRI, and the 425-bp fragment was ligated to EcoRI-digested pCU1 (Augustin et al., 1992). A plasmid with the expected restriction pattern, where the 425-bp fragment can be excised by digesting with EcoRI and not with SphI, was identified and designated pNB014. This plasmid was digested with SphI and HindIII, and the resulting 5.6-kbp fragment was ligated to the annealed complementary oligonucleotides jc9 and jc10, encoding for the mersacidin peptide with a silent mutation to create a BsrGI site. Plasmids containing the newly introduced BsrGI site were selected and sequenced. The plasmid with the expected sequence was designated pNB018. This plasmid is a pCU1 derivative containing both the structural gene *mrsA* and its promoter, modified such that the sequence encoding for mersacidin can be removed or substituted. Digestion with SphI and BsrGI enables the residues from 1 to 12 to be modified, whereas digestion with BsrGI and HindIII allows the modification of residues 12–20. Multiple alterations of the entire region encoding mersacidin can be introduced through digestion with SphI and HindIII.

### Complementation of *Bacillus* Sp. TTEX

A complementation test of *Bacillus* sp. TTEX with plasmid pNB018 was performed to assess the production of mersacidin when *mrsA* is complemented *in trans*. Plasmid pNB018 was used to transform *Escherichia coli* ET12567 to obtain demethylated plasmid. Electrocompetent *Bacillus* sp. TTEX was transformed with this plasmid, and selected transformants were grown and tested for mersacidin production. *Bacillus* sp. TTEX did not produce mersacidin, *Bacillus* sp. HIL Y-84,54728 produced 140–160 mg/l mersacidin, and the complementation mutant produced 60–80 mg/l mersacidin. These results showed that mersacidin production was restored in *Bacillus* sp. TTEX when transformed with pNB018, indicating that the complementation system is a viable method for producing mersacidin variants.

### Construction of a Saturation Mutagenesis Library of Residues Not Involved in Bridge Formation

**Figure 2:**
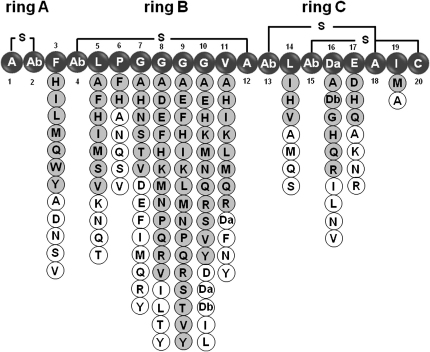
Mersacidin Substitution Library Under the linear representation of mersacidin, the circles indicate the variants that were detected by LC-MS. Gray circles indicate variants produced at high yield (>10%, versus mersacidin production), and white circles indicate variants produced at trace levels. Da, dehydroalanine; Db, dehydrobutyrine; Ab, 2-aminobutyrate.

The pattern of lanthionine bridges of mersacidin is conserved in other lantibiotics, such as actagardine (Zimmermann et al., 1995), michiganin A (Holtsmark et al., 2006), lacticin 3147-A2 (Ryan et al., 1999), plantaricin Wα (Holo et al., 2001), staphylococcin C55α (Navaratna et al., 1998), haloduracin (McClerren et al., 2006), and lichenicidin (Rey et al., 2004), suggesting that it might contribute to the folding required for binding lipid II. Taking this into consideration, a saturation mutagenesis library was designed, keeping the residues involved in bridge formation unchanged. Mutant plasmids targeting the 12 amino acids that are not involved in lanthionine bridges were constructed (Figure 1), making a collection of 228 (12 × 19) plasmids. Individual plasmids were used to transform *Bacillus* sp. TTEX. Five colonies from each transformation were grown and analyzed for production of the corresponding mersacidin variant. Production of the variants was evaluated by liquid chromatography-mass spectrometry (LC-MS) and by antibacterial activity against *Micrococcus luteus.* Figure 2 shows the outcome of the analysis of fermentation samples of the 228 mutants. From this collection of mutants, 82 were expressed and processed through to mature mersacidins at high yields, as evidenced by detection of an ion with the corresponding mass for a fully processed mersacidin analog (Figure 2). A set of 48 variants was produced at trace level and was not further purified for antimicrobial evaluation (Figure 2). Production of mersacidin variants was detected for each position tested, but some positions, such as I19, E17, L14, and P6, are less permissive, particularly I19, where only the variant I19M was produced at high yield. The central part of ring B of mersacidin, from residues 7 to 11, is highly permissive to substitutions allowing an average of more than 10 changes per residue.

Interestingly, mutants with cysteine replacing any of the residues in the mersacidin molecule were not detected. It is possible that the introduction of a new cysteine would have led to scrambling of the ring structures, but there was no clear evidence by LC-MS for production of molecules with altered numbers of ring structures. In contrast, mutants incorporating serine or threonine were produced at a number of positions. Serines or threonines incorporated at positions 5 to 9 did not appear to be dehydrated (though formally it cannot be ruled out that these positions were dehydrated and other normally dehydrated moieties were not), and these results agree with the finding that glycine residues adjacent to serine in the lantibiotic lacticin 481 impair the dehydration reaction of LctM in vitro (Chatterjee et al., 2006). In contrast, both serine (introduced at position 10 or 11) and threonine (introduced at position 10) were partially dehydrated. When threonine was substituted for the normally dehydrated serine at position 16, this appeared to be fully dehydrated.

### Substitution of the Threonines Involved in Ring Formation by Serines

Mutants in which the threonine residues involved in ring formation (positions 2, 4, 13, and 15) were substituted by serine were constructed. Analysis of fermentation samples of these mutants by LC-MS showed no evidence of lantibiotic production. These types of substitutions have been productive for several lantibiotic systems, including type I and type II classes, so it is surprising that the mersacidin pathway was not permissive for any of the four possible mutants. The dehydration of serine would produce dehydroalanine, which is more reactive than dehydrobutyrine, and in this system, it might form unnatural bridging patterns by spontaneously reacting with cysteine. In this case, as with all the other nonproductive mutants, it cannot be ruled out that the new molecules are unable to autoinduce the biosynthetic pathway, leading to very low or undetected amounts of the respective lantibiotic variant being produced.

### Construction of an Insertion Library

**Figure 3:**
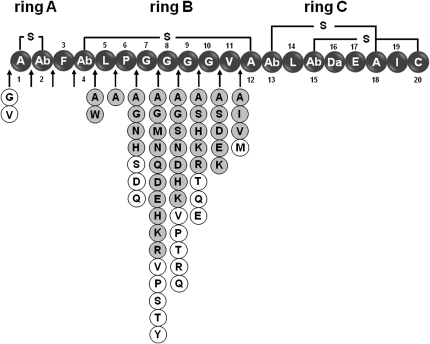
Mersacidin Insertion Library Under the linear representation of mersacidin, the arrows indicate the positions where residues were inserted, and the circles indicate the insertion variants that were detected by LC-MS. Gray circles indicate variants produced at high yield (>10%, versus mersacidin production), and white circles indicate variants produced at trace levels. Da, dehydroalanine; Db, dehydrobutyrine; Ab, 2-aminobutyrate.

An insertion library was constructed wherein each of the 20 possible amino acids was inserted at the N terminus and between each pair of residues up to the end of ring B, between V11 and C12 of mersacidin, making a collection of 240 plasmids (12 × 20) (Figure 3). There were no attempts to introduce residues in ring C, because the size and sequence of this ring is conserved in this class of lantibiotics and is believed to be involved in binding to lipid II, although the presence of the equivalent ring in haloduracin is not required for antibacterial activity (Cooper et al., 2008). Individual plasmids were used to transform *Bacillus* sp. TTEX. Five colonies from each transformation were grown and analyzed for production of the corresponding variant and for antibacterial activity. Figure 3 shows the outcome of the analysis of fermentation samples of the 240 mutants. From this collection of mutants, 37 were expressed and processed through to mature mersacidins at high yields, as evidenced by detection of an ion corresponding to the expected mass for a fully processed mersacidin analog (Figure 3). Again, evidence was found for production of other variants at much lower levels. The N terminus of mersacidin was extended with glycine or valine, but the new variants were produced only at trace levels. None of the 20 amino acids could be inserted into ring A, indicating that the biosynthetic machinery is specific for a two-membered ring. Furthermore, the region between rings A and B could not be extended with any of the insertions attempted. The center of ring B was the most permissive area, as in the substitution library. Insertions of polar and nonpolar residues were well tolerated without an obvious pattern of preference regarding chain length or charge, apart from the fact that neither proline nor aromatic amino acids were accepted, with the exception of tryptophan between positions 4 and 5. As with the substitution library, cysteine was not incorporated. Serine was incorporated after amino acids 8, 9, or 10 but did not appear to be dehydrated. This is in contrast to the G10S or V11S mutants in which the newly introduced serine was partially dehydrated.

### Construction of Deletion Mutants

A collection of deletion mutants for each position within ring B (positions 5–11) of mersacidin was constructed. Again, no deletions were attempted on ring C as it is believed to be involved in lipid II binding. Individual plasmids were used to transform *Bacillus* sp. TTEX. Five colonies from each transformation were grown and analyzed for production of the corresponding variant. The processing enzymes do not appear to tolerate any reduction in the size of ring B, as no mature lantibiotics were detected following LC-MS analysis of fermentation samples.

### Antibacterial Activity of Mersacidin Variants

Partially purified fermentation samples from clones expressing highly produced variants were assayed for antimicrobial activity against *M. luteus*. Samples were fractionated to eliminate interfering bioactive compounds produced by the host strain and were bioassayed (see Supplemental Data available online). Selected mersacidin variants with antibacterial activity against *M. luteus* were produced at larger scale for quantitative studies (2 l). The mersacidin variants were purified and assayed against a panel of microorganisms to determine the MIC values. Table 1 shows the activity of selected compounds compared with that of mersacidin.

All mersacidin variants with amino acid insertions showed poor activity, compared with mersacidin. Eight variants showing activity in preliminary screening against *M. luteus* were purified for MIC determination, but none of these variants was active against the target strains at a concentration of 64 μg/ml (data not shown).

In contrast, many of the substitution variants retained significant antibacterial activity. Mersacidin variants with antibacterial activity are listed in Table 1. Some variation to the molecule was tolerated at all of the positions tested except for L5, E17, and I19. In most of the cases, mutants retaining appreciable activity contained conservative amino acid changes, though some nonconservative changes were tolerated in ring B (e.g., G7N, G8Q, G9H, G10Y, and even P6H).

The variant F3W showed higher activity than did mersacidin against most of the organisms tested. Variants G9A and G9H showed activity comparable to that of mersacidin against *Staphylococcus*, but lower activity against *Streptococcus pneumoniae* and enterococci. Variants V11I, L14I and S16Db also showed activity comparable to that of mersacidin against *Staphylococcus.* L14I and S16Db also retained activity against enterococci, whereas V11I had improved activity against these strains. L14I also had promising activity against *S. pneumoniae.*

Representative variants with activity better than or comparable to that of mersacidin were selected, and double mutants combining the favorable mutations were constructed. The double mutants F3W-L14I and V11I-L14I had activities comparable to those of the parent mutants F3W and V11I, respectively, but no additive effect was found.

## Discussion

Several studies have been performed to manipulate the structure and biological activity of lantibiotics by site-directed mutagenesis of the *lanA* genes (Cotter et al., 2005). Specific mutations for different lantibiotics are well tolerated in that new fully processed lantibiotics are synthesized, but others result in partially processed lantibiotics or abolish production altogether. The first systematic mutant analysis of a lantibiotic was performed using the two-component lantibiotic lacticin 3147 (Cotter et al., 2006). Alanine-scanning mutagenesis of this lantibiotic suggests that there are particular areas within the peptides that are amenable to changes and areas that are essential for production of the final compound. Extensive investigations have also been performed over a number of studies involving the lantibiotic nisin with similar results (Rink et al., 2007b; Field et al., 2008).

The present study represents the most extensive and systematic exploration to date of the ability of the lantibiotic biosynthetic machinery to tolerate changes to the primary amino acid structure of the propeptide. When a saturation mutagenesis library of 228 mersacidin mutants was analyzed, we found that over 80 mutants produced mature mersacidin variants at good levels (Figure 2). Undoubtedly, other mutants were produced, albeit at lower production levels. This finding indicates a remarkable flexibility in the lantibiotic biosynthetic machinery. In contrast, the overall geometry of the lantibiotic appears to be under stricter control. No deletions of amino acids in ring B were tolerated, suggesting that this ring may have a minimum size. The equivalent ring B of actagardine has six residues, compared with nine for mersacidin, suggesting that the constraint may lie with the requirements of the processing enzymes and not in the premersacidin peptide. Furthermore, no insertions into ring A or between ring B and ring C were tolerated. Although insertions into ring B were well tolerated, the number and range of amino acids accepted was not as high as for substitutions, and production of insertion variants decreased in positions close to the lanthionine bridge.

Ring B, especially amino acids 7–11, constitute a “hypervariable region” similar to the one identified in lacticin 3147-A2 for the equivalent ring during alanine-scanning studies (Cotter et al., 2006). As might be expected, conservative changes were mostly tolerated, but nonconservative changes were also successful. Indeed, other than the nonacceptance of cysteine, there was little obvious pattern in terms of the acceptance or otherwise of amino acid substitutions.

Analysis of 37 lantibiotic primary structures in a study by Rink et al. (2005) showed that dehydratable serine and threonine residues are more often flanked by hydrophobic (nonaromatic) amino acids than by hydrophilic amino acids, and serine residues more often escape dehydration than threonine. These findings have been experimentally supported for the nisin system (Rink et al., 2007a). In the mersacidin system, the outcome supports this study but only partially. At position 3 (flanking two dehydrated amino acids), the hydrophobic amino acids isoleucine and leucine and aromatic amino acids were accepted as well as methionine, glutamine, and histidine, which are very rarely seen as flanking amino acids in natural lantibiotics. Similarly, at position 5, as well as the “expected” A, I, and V substitutions, the residues M, S, F, and H were well tolerated. Other than F, these amino acids are rarely seen C-terminal to dehydrated amino acids in natural lantibiotics, suggesting that the mersacidin modification enzymes might have different substrate specificities. At position 11, at the N-terminal side of a cysteine residue, the results were more in line with the findings of Rink et al. (2005). Perhaps it is important to point out that the consensus of Rink et al. (2005) was obtained using class I and class II lantibiotics combined, and the dehydratases for these two classes have no sequence homology. For class II enzymes, such as LctM, it has been reported that negatively charged flanking residues do not prevent dehydration (Chatterjee et al., 2006). For mersacidin in particular, the multiple concatenated rings in the ring C part of the molecule can make interpretation rather complex.

With regard to dehydration of introduced serines and threonines, no difference between the two residues was noted except that introduction of serine was perhaps more generally tolerated. With the exception of position 16, where there is naturally a dehydrated serine residue and an introduced threonine is fully dehydrated (in poor concordance with the flanking amino acid consensus of Rink et al., 2005), introduced serine or threonine residues were not fully processed at high yield. Interestingly, none of the ring-forming threonines could be replaced by serine, suggesting high specificity for either the dehydration or ring-forming machinery, although dehydration of serine at position 16 suggests that there is no specificity for threonine during dehydration. As mentioned above, the more reactive dehydroalanine might form lanthionine bridges spontaneously with cysteine residues, producing prepeptides that could inhibit any further step of the pathway. It is surprising that these substitutions were not productive since equivalent changes are tolerated in nisin (Dodd et al., 1992) and in the in vitro synthesis system of lacticin 481(Chatterjee et al., 2006). Regarding the subsequent steps of the pathway, the specificity of LctT (Furgerson Ihnken et al., 2008) or NisT (Kuipers et al., 2004) do not seem to be limiting steps so it is not expected to be for mersacidin. The secretion and processing of misfolded MrsA mutants might take place, but the accumulation of the final peptide in the medium might be limited because of the action of proteases produced by the host strain in these fermentation conditions. The development of an in vitro synthesis system of mersacidin should clarify this finding and will help to establish unequivocally whether MrsM can differentiate serine-to-threonine substitutions in these positions.

It appears that there are no simple rules to govern amino acid acceptance. They may be different for each lantibiotic and modification enzymes and they may not be understandable from the primary amino acid sequence but may require examination of the secondary and tertiary structure of the peptides.

Interestingly, where no mature lantibiotic was observed, no evidence was found for significant levels of partially processed peptides. If the introduction of an amino acid causes failure of a dehydration or cyclization step, it may be that the “malformed” peptide fails to act as an inducer for the biosynthetic apparatus because mersacidin induces its own production (Schmitz et al., 2006). This could be avoided by adding mersacidin to the production medium but would complicate the evaluation of the biological activity of the newly produced variants because some would copurify with mersacidin. Alternatively, molecules that are not fully cyclized may fail to be exported, and their leader peptides might not be processed or may fall subject to proteolysis, because *Bacillus* sp. are prolific producers of proteases.

Indeed, apart from MrsM, which is responsible for lanthionine formation, other enzymes implicated in tailoring (MrsD), cleavage and export (MrsT), immunity, and regulation may constitute further barriers to lantibiotic engineering. Van der Meer et al. (1994) showed that, for the nisin biosynthetic machinery, dehydration and export were remarkably tolerant of amino acid variation but that the leader sequence cleavage was quite specific. A recent breakthrough in the in vitro synthesis of the lantibiotic lacticin 481 using the isolated LctM enzyme offers the potential to look directly at individual steps of the lantibiotic biosynthetic pathways and to understand the parameters that control the ability to manipulate the lantibiotic structure (Xie et al., 2004). In this context, the low number of variants obtained at position I19 might be explained by its proximity to C20, because this residue is the substrate for the oxidative decarboxylation reaction of MrsD, the first step in the modification of MrsA (Majer et al., 2002). It seems likely that the specificity of MrsD limits the productivity of variants at this position. No insertions were well tolerated at the N terminus, probably as the result of interference with processing of the leader peptide or, possibly, as the result of subsequent proteolysis. We are currently attempting to establish an in vitro system for mersacidin biosynthesis. Only in vitro studies will ultimately allow dissection of the roles of different elements of the biosynthetic machinery and, eventually, the overcoming of intrinsic limitations, perhaps by mutation and evolution of the biosynthetic machinery.

With regard to the activity of mersacidin variants, it was apparent that any change in the overall geometry of the lantibiotic-like insertion of new amino acids essentially abolished antibacterial activity. It would appear from this result that the overall geometry of the molecule has been evolved for a very precise interaction with lipid II. However, it is interesting in this context that other lipid II-binding lantibiotics of the mersacidin class have profoundly different overall geometries, with only ring C as the highly conserved part of the molecule. Given the high degree of conservation of ring C in the mersacidin class of lantibiotics, it is not surprising that only conservative changes at position L14 produced compounds with activity comparable to that of mersacidin, because this position is highly conserved. Tolerance to changes at position 16 would be less surprising because this position does seem to vary, being dehydroalanine for mersacidin, I for actagardine, H for lacticin 3147, and K for lichenicidin. Our results concur with the findings of Szekat et al. (2003) in that the glutamate at position 17 appears to be essential for activity. The equivalent Glu residue in haloduracin has been substituted for alanine or glutamine, and antibiotic activity is abolished (Cooper et al., 2008). Regarding the conservation of ring C in this type of lantibiotic, it has been shown that when the equivalent ring in lacticin 3147 was opened, bioactivity was abolished (Cotter et al., 2006; Field et al., 2007), but for haloduracin, the compound was synthesized and surprisingly maintained antibacterial activity (Cooper et al., 2008). This study suggests that the sequence conservation might be important for binding lipid II but that the lanthionine bridge is not essential for bioactivity; perhaps the folding of the final products might be different when they are synthesized in vivo, or the contribution of the second peptide in the folding of the final antibiotic might be important for this ring. Significantly, the results presented here do indicate that it is possible to obtain novel mersacidin variants with improved antibacterial activity by engineering of the primary amino acid structure. Variant molecules with improved overall bioactivity, such as F3W, offer significant potential for development of therapeutic agents.

## Significance

**This study is the most extensive mutagenesis analysis performed on a lantibiotic and was achieved using an efficient *trans* complementation system to generate mersacidin mutants in the producer strain. A high number of new mersacidin molecules were generated showing that the biosynthetic pathway of this lantibiotic has relaxed specificity. Ring A of mersacidin and its flanking residues cannot be extended. Ring B is particularly amenable to substitutions and insertions with conserved and nonconserved residues, but the positions adjacent to the bridge-forming residues within this ring tend to be more stringent. Surprisingly, deletions were not tolerated in ring B. Mersacidin contains methyl-lanthionine bridges only, and mutants encoding for mersacidin variants with lanthionine bridges were not productive. The antibacterial activity of the new molecules was assessed, and most of the newly formed compounds were inactive or showed less potency than did mersacidin, but compounds with improved activity against different Gram-positive pathogens were identified, suggesting that an improvement program in this type of compounds can be performed using this methodology.**

## Experimental Procedures

### Strains, Plasmids, Oligonucleotides, Culture Conditions, and DNA Manipulations

The strains, plasmids, and oligonucleotides used for genetic manipulations and production of mersacidins are described in Table 2. *E. coli* cultures were grown at 37°C in Luria broth (LB) or Luria agar (LA) supplemented with the appropriate antibiotic. *Bacillus* cultures were grown in tryptic soy broth (TSB), tryptic soy agar (TSA), or LA at 30°C supplemented with the appropriate antibiotic. The composition of mersacidin production medium is 50 mM (NH_4_)_2_SO_4_, 2 mM MgSO_4_, 1 mM CaCl_2_.7H_2_O, 0.2 mM FeSO_4_.7H_2_O, 1 mM MnSO_4_.H_2_O, 1 M potassium phosphate buffer (pH 7.0; 40 ml/l); 1 M Tris maleate buffer (pH 7.0; 100 ml/l), and 400 mM glucose added after sterilization. PCR was performed using a Robocycler Gradient 96 (Stratagene) and Pfu polymerase (Promega). The reaction conditions were as follows: Cycle 1, 95°C for 3 min, 45°C for 1 min, and 72°C for 1 min; and cycles 2–26, 95°C for 1 min, 45°C for 1 min, 72°C for 1 min, and a further incubation step at 72°C for 10 min. Site-directed mutagenesis to obtain plasmids encoding for mersacidin variants was performed using the method ExhauSeq (Delcourt, 2006) together with pNB018 as a template.

### Electroporation of *Bacillus* Sp. TTEX

*Bacillus* sp. TTEX was transformed by electroporation using a modification of the method described by Xue et al. (1999). *Bacillus* sp. TTEX was used to inoculate 10 ml of LB, and the culture was incubated at 30°C and 250 rpm for 16 hr. An aliquot of 3 ml of this culture was used to inoculate 50 ml of TSB with sorbitol and mannitol (both to 0.5 M final concentration) in a 250-ml conical flask. This culture was grown at 30°C and 250 rpm for 4.5 hr (OD_600_ = 2.0). The culture was cooled on ice for 10 min before centrifuging at 2,500 rpm for 30 min. The cell pellet was resuspended in 6 ml of ice-cold electroporation medium (10% glycerol in 1 M sorbitol, and 0.75 M mannitol) and was centrifuged for 3 min at 13400× g. The cells were washed three times with 3 ml of ice-cold electroporation medium (with centrifugation for 3 min at 13400× g) before finally resuspending in 1 ml of ice-cold electroporation medium. Aliquots of 60 μl were used for electroporation, either immediately or after storage at −80°C. Demethylated plasmid DNA (500–1000 ng in 1.5 μl) obtained from *E. coli* ET12567 was added to electrocompetent cells and transferred to a precooled 1 mm gap electroporation cuvette. The voltage used was 2500 V on an electroporator preset for 10 μF capacitance and 600 Ω resistance. Following electroporation, 1 ml of recovery medium was added (TSB with 1 M sorbitol and 0.75 M mannitol final concentration), and the suspension was transferred to a 1.5-ml centrifuge tube and incubated at 30°C for 3 hr. After centrifugation at 13400× g for 3 min, the pellet was resuspended in 200 μl of TSB and plated on LA containing the appropriate antibiotic for selection.

### Fermentation Conditions of *Bacillus* Sp. HIL Y-84,54728 and Mutants for Production of Mersacidin

*Bacillus* sp. HIL Y-84,54728 or mutant colonies were transferred to 15-ml culture tubes containing 3 ml of TSB (supplemented with 25 mg/l chloramphenicol [Cm] for complementation mutants). These seed cultures were incubated at 30°C and 250 rpm for 24 hr; an aliquot of 0.5 ml of each culture was used to inoculate 50-ml conical flasks containing 10 ml of mersacidin production medium (supplemented with 25 mg/l Cm for complementation mutants). The cultures were incubated at 30°C and 250 rpm for 5 days. For large-scale fermentations, seed cultures were grown in 250-ml conical flasks containing 50 ml of TSB with 25 mg/l Cm. Cultures were incubated at 250 rpm and 30°C for 24 h, an aliquot of 20 ml of seed culture was used to inoculate 2-l conical flasks containing 500 ml of production medium with 25 mg/l Cm. The cultures were incubated at 30°C and 250 rpm for 5 days.

### Detection and Evaluation of Mersacidin by Bioactivity

Antibacterial activity of fermentation samples or purified samples was determined by Mueller-Hinton Agar diffusion bioassays using *M. luteus* ATCC 4698 as the indicator strain. Susceptibility testing (minimum inhibitory concentration [MIC]) for all aerobic organisms, with the exception of *S. pneumoniae*, was performed following the recommendations of CLSI. Susceptibility testing of *S. pneumoniae* was performed by two-fold serial dilutions in brain-heart-infusion broth supplemented with 50 μg/ml Ca^2+^. Vancomycin was used as a reference antibiotic, and plates were incubated aerobically, with shaking, for 18–20 hr at 37°C with the MIC defined as the lowest concentration of drug that prevented visible growth.

### Detection of Mersacidin Variants by LC-MS

Fermentation samples were spun down at 3450× g for 10 min in 15-ml centrifuge tubes. The supernatants were transferred to 50-ml centrifuge tubes containing 100 mg of conditioned resin Diaion HP-20 (Supelco). After incubation at room temperature for 6 h with shaking, the supernatants were discarded, and the resin containing the bound mersacidin variant was washed twice with 10 ml of water. Two further washing steps were performed with 10 ml of methanol:water (1:1). Mersacidin variants were eluted from the resin with 1 ml of 100% methanol. The eluates were dried down, resuspended in 250 μl of methanol:water (1:1), and analyzed by LC-MS, high-performance liquid chromatography (HPLC), and bioassay. Samples of 20 μl were analyzed by LC-MS using the HPLC gradient conditions listed in Supplemental Data. Mass spectrometry conditions and the procedure for fractionation of fermentation samples to eliminate background antibiotic activity are listed in Supplemental Data. Mersacidin variants were isolated using the methods described in Supplemental Data.

## Figures and Tables

**Table 1:** In Vitro Activity of Mersacidin and Substitution Variants

Compound	*S. aureus* SH1000^a^	*S. aureus* R33 MRSA	*S. epidermidis* NCTC11047	*S. pneumoniae* BAA-255	*M. luteus* ATCC4698	*E. faecium* ATCC19579	*E. faecalis* ATCC29212	*E. faecium* 7131121 VRE^b^
Mersacidin	32	32	16,32	2	1,2	32	64	64
F3W	16	8	8	2,4	0.5	16	32	32
P6H	64	32,64	32,64	64, >64	16	>64	>64	>64
G7A	>64	32,64	64	>64	64	64	>64	>64
G7N	64	16,32	32	16	< 4	64	>64	>64
G8H	64, >64	64	64	16	8	>64	>64	>64
G8N	>64	16,32	64	>64	8	>64	>64	>64
G8Q	>64	32	32,64	32,64	8	>64	>64	>64
G9A	32	8	32	8	< 4	64	>64	>64
G9H	32	8,16	32	16	< 4	64	>64	>64
G9S	>64	64, >64	>64	>64	64	>64	>64	>64
G10A	>64	64, >64	64, >64	8	8	>64	>64	>64
G10N	>64	64, >64	>64	>64	8	>64	>64	>64
G10V	>64	64, >64	64	16	< 4	>64	>64	>64
G10Y	>64	64	8,16	< 4	< 4	64	32	>64
V11I	32	16	16	16	0.5	16	16	16
V11L	64	32	16	4,8	4,8	32	64	32
V11M	64	32	16,32	32	16	32	64	64
L14I	32	8	8,16	1	1,2	16	32	32,64
L14M	32	16	16,32	4	2	64	64	64
L14V	32	16	16	4	4,8	64	64	>64
S16A	64	16	32	4	2	32	64	>64
S16Db	32	8	16	32	1	32	32	64
S16G	32	16	16	2	2,4	32	64	64

MIC data are in μg/ml.

a Horsburgh et al. (2002).

b Vancomycin-resistant clinical isolate.

**Table 2:** Strains, Plasmids, and Oligonucleotides Used in This Work

Strain or plasmid	Use	Source
*Bacillus* sp. HIL Y-8,54728	Mersacidin producer	Chatterjee et al., 1992a
*Bacillus* sp. TTEX	Host for mersacidin library	Schmitz et al., 2006
*Escherichia coli* DH10B	Cloning host	Invitrogen
*E. coli* ET 12567	Host for demethylated DNA	MacNeil et al., 1992
*Micrococcus luteus* ATCC4698	Bioassay for mersacidins	ATCC
pMER1	Template for PCR	Altena et al., 2000
pUC18	*E. coli* cloning vector	Yanisch-Perron et al., 1985
pCU1	*E. coli-Bacillus* shuttle	Augustin et al., 1992
pNB013	Intermediate for *mrsA* expression	This work
pNB014	Intermediate for *mrsA* expression	This work
pNB018	*mrsA* and variants expression	This work

Oligonucleotide	Sequence (5′-3′)
Jc7	CTTATGAGAATTCGAGACAAGGTAAACT
Jc8	GCATGCTGCTTCCATGTCTCCCGCACCTACT
Jc9	CACTTTTACATTGCCTGGTGGCGGCGGTGTTTGTACACTAACTTCTGAATGTATTTGTTA
Jc10	AGCTTAACAAATACATTCAGAAGTTAGTGTACAAACACCGCCGCCACCAGGCAATGTAAAAGTGCATG

## Supplemental Data

Document S1. Three Tables
