# Supplementary material for: Dissecting Structural and Functional Diversity of the Lantibiotic Mersacidin
Source: Chem Biol. 2009 May 29;16(5-3):490–8. doi: 10.1016/j.chembiol.2009.03.011 (PMC2706954; doi:10.1016/j.chembiol.2009.03.011)
Supplement: Document S1. Three Tables [file mmc1.pdf]

Supplemental Data

Dissecting Structural and Functional Diversity of the Lantibiotic Mersacidin

Antony N. Appleyard, Shaila Choi, Daniel M. Read, Ann Lightfoot, Steven Boakes, Anja Hoffmann, Ian Chopra, Gabriele Bierbaum, Brian A.M. Rudd, Michael J. Dawson, and Jesus Cortes

Analytical methods for mersacidin and its variants

Table S1. HPLC Conditions Used in the Analysis of Fermentation Samples and Fermentation Concentrates by LC-MS

Column	Phenomenex Luna HPLC column (5 µ, C18(C2), 150 × 4.6 mm)		
Mobile Phase A	10 % Acetonitrile / 0.1 % Formic Acid		
Mobile Phase B	90 % Acetonitrile / 0.1 % Formic Acid		
Flow Rate	1 ml/min		
Gradient	Time 0 minutes	100 % A	0 % B
	Time 10 minutes	0 % A	100 % B
	Time 11 minutes	0 % A	100 % B
	Time 11.1 minutes	100 % A	0 % B
	Time 15 minutes	100 % A	0 % B
	Cycle time 15 minutes		

Table S2. Mass Spectrometer Parameters Used in the Analysis of Broth Samples and Fermentation Concentrate by LC-MS

Ionisation	Electrospray +ve mode
Mass Range	250 to 1500 m/z
Capillary Voltage	3.10 KV
Cone Voltage	40 V
Skimmer Lens Offset	5 V
Ion Energy	1.4

Partial purification of Fermentation Samples for Antibacterial Evaluation

Prior to bioassay the broth samples and fermentation concentrates, samples were fractionated using an analytical HPLC coupled to a 96 well microtitre plate fraction collector. In general, 0.200 ml of broth sample or fermentation concentrate was loaded onto the column and the components were resolved and collected as described in Table 3. The fractions in the 96 well microtitre plates were evaporated to dryness and the resulting residues were dissolved in 50 µl of methanol:water (1:1). For each variant the resuspended residues from fractions 36 to 43 were loaded onto bioassay agar plates containing *Micrococcus luteus* ATCC 4698 as indicator strain. The bioassay plates containing the mersacidin variants samples were left at room temperature for 1 hour to allow diffusion of the sample into the agar prior to incubation at 30 ºC overnight.

Table S3. Analytical HPLC Conditions Used to Fractionate Fermentation Samples and Fermentation Concentrates

Column	Phenomenex Luna HPLC column (3 µ, C18(C2), 150 × 4.6 mm)
Mobile Phase A	30 % Acetonitrile
Mobile Phase B	65 % Acetonitrile

Flow Rate	1 ml/min		
Gradient	Time 0 minutes	100 % A	0 % B
	Time 10 minutes	0 % A	100 % B
	Time 11 minutes	0 % A	100 % B
	Time 11.2 minutes	100 % A	0 % B
	Time 15 minutes	100 % A	0 % B
	Cycle time 15 minutes		
Injection Volume	200 µl		
Detection	254 and 210 nm		
Fraction Collection	0.2 min/fraction		
Fractions Collected	60 fractions		
